# Value of Strain-Ratio Elastography in the Diagnosis and Differentiation of Uterine Fibroids and Adenomyosis

**DOI:** 10.3390/jpm11080824

**Published:** 2021-08-23

**Authors:** Vladut Săsăran, Sabin Turdean, Marius Gliga, Levente Ilyes, Ovidiu Grama, Mihai Muntean, Lucian Pușcașiu

**Affiliations:** 1 Department of Obstetrics and Gynecology 2, Faculty of Medicine in English, “George Emil Palade” University of Medicine, Pharmacy, Sciences and Technology of Târgu Mureș, Gheorghe Marinescu Street No. 38, 540136 Târgu Mureș, Romania; vlad_sasaran0403@yahoo.com; 2 Department of Morphopathology, Faculty of Medicine, “George Emil Palade” University of Medicine, Pharmacy, Sciences and Technology of Târgu Mureș, Gheorghe Marinescu Street No. 38, 540136 Târgu Mureș, Romania; 3 Department of Obstetrics and Gynecology 2, Faculty of Medicine, “George Emil Palade” University of Medicine, Pharmacy, Sciences and Technology of Târgu Mureș, Gheorghe Marinescu Street No. 38, 540136 Târgu Mureș, Romania; m11gliga@yahoo.com (M.G.); iliasz2001@yahoo.com (L.I.); ovi_grama@yahoo.com (O.G.); 4 Department of Obstetrics and Gynecology, Clinical County Hospital Mures, Samuel Koteles Street No. 29, 540057 Târgu Mureș, Romania; munteanmihai@yahoo.com; 5 Department of Obstetrics and Gynecology 1, Faculty of Medicine in English, “George Emil Palade” University of Medicine, Pharmacy, Sciences and Technology of Târgu Mureș, Gheorghe Marinescu Street No. 38, 540136 Târgu Mureș, Romania; puscasiu@gmail.com

**Keywords:** adenomyosis, uterine fibroids, transvaginal ultrasound, strain ratio elastography

## Abstract

Benign uterine disorders, including uterine fibroids (UF) and adenomyosis (AM), can impact the life quality and fertility of women of reproductive age. Transvaginal ultrasound (TVUS) has long been used for their early identification, but its combined use with elastography seems to improve diagnostic accuracy of UF and AM. Thus, a prospective pilot study was conducted on 79 patients who underwent hysterectomy (25 microscopically diagnosed with AM and 53 with UF), with the aim of assessing the ability of TVUS combined with strain ratio elastography (SE) to accurately diagnose and distinguish UF and AM. Significantly higher mean and maximal strain ratio (SR) values were identified for patients with histologically confirmed AM as opposed to those with UF (*p* < 0.001). Diagnostic sensitivity and specificity, calculated in comparison with histology results, were higher for UF than AM. Receiver operating characteristic (ROC) analysis was applied between the two study groups, revealing cutoff values of 7.71 for mean SR and 8.91 for maximal SR, respectively, with good sensitivity and specificity parameters (100% and 96.23%; 96% and 96.23%). Our results support the use of TVUS in combination with SE for the positive and differential diagnosis of UF and AM, through identification of their particular tissue stiffness features.

## 1. Introduction

Adenomyosis and uterine fibroids represent two benign distinct uterine disorders, with an important frequency among young women [1]. Adenomyosis (AM) is defined by abnormal migration of endometrial tissue into the myometrium and causes myometrial inflammation and hypertrophy [2,3]. Its finding among uterine specimens obtained after hysterectomy vary between 5 and 70%, and the prevalence of this pathology among the general population is considered to be 28.9 in 10,000 women [2,4]. These abnormal modifications greatly impact quality life in women of reproductive age, leading to dysmenorrhea, pelvic pain, abnormal uterine bleeding and infertility [3]. Its diagnosis is often problematic, as the frequent association between adenomyosis, uterine fibroids and endometriosis is responsible for an unspecific clinical pattern [4]. Furthermore, early identification of adenomyotic lesions might be strongly influenced by their localization, as they may involve only few areas of the myometrium, or, in some cases, the entire uterine wall, extending from focal to diffuse adenomyosis [5]. Thus, the diagnosis of adenomyosis remains a continuous challenge, in spite of considerable improvement in non-invasive diagnostic techniques. Still, histopathological examination currently remains the gold standard in the diagnosis of adenomyosis [6]. Sharing common symptoms with adenomyosis, uterine fibroids (UF) represent one of the most common pathologies in women during reproductive years, with an incidence among the general population of up to 70% [1,7]. Characterized by heterogeneity throughout the years, these tumors are also known as leiomyomas and are usually diagnosed incidentally, remaining asymptomatic for prolonged periods of time [7,8]. Symptomatic fibroids will cause compression symptoms (urinary incontinence and constipation) due to their size and location, abnormal uterine bleeding, sexual dysfunction, dysmenorrhea and infertility [7].

Transvaginal ultrasound (TVUS) is one of the most valuable tools in gynecology, being frequently used in differential diagnosis of gynecological pathologies. TVUS represents the first line imaging approach in gynecological clinical practice, as it is cheap, non-invasive and relatively precise when used by an experimented examinator [9]. MUSA (Morphological Uterus Sonographic Assessment) group criteria, which comprised the assessment of uterine corpus (symmetry and echogenicity), presence of myometrial and endometrial lesions and their characteristics (number, location, site, size and shadowing), and vascularity of myometrium improved 2D TVUS accuracy in diagnosis of AM and UF [10]. Elastography in combination with TVUS, a new imaging tool which delivers information about lesion and surrounding tissue stiffness, was another method used for the diagnosis of these two benign uterine pathologies [3,11,12]. Two systems are frequently used, shear wave elastography (SWE), a quantitative method which provides information about tissue stiffness, and strain ratio elastography (SE), a qualitative method which compares one tissue stiffness with another [12]. Both methods have been regarded as potential valuable assets in combination with TVUS for the diagnosis of benign uterine pathologies, as well as for the distinction between benign and malignant uterine lesions [13,14,15]. In particular, abnormal modifications of tissue elasticity in AM and UF lead to imaging variations in SE, which may increase the diagnosis accuracy of this pathologies [3]. Still, a consensus is lacking regarding the utility and accuracy of this combined method in the preoperatory diagnoses of several uterine pathologies, as compared to other imaging methods [15].

The aim of this study is to evaluate TVUS and SE accuracy in detecting AM and to assess its utility in differentiating AM from UF.

## 2. Materials and Methods

We conducted a pilot, prospective study in the Clinical County Hospital of Mures between May 2019 and May 2021.

### 2.1. Study Population and Groups

We enrolled 79 patients who underwent surgical treatment for suspicions of two benign uterine disorders, adenomyosis and uterine fibroids (leiomyoma), raised upon 2D TVUS in combination with SR elastography. Symptoms of patients included in the study consisted of chronic pelvic pain, dysmenorrhea, treatment-resistant menometrorrhagia and compression symptoms due to uterine size. Each patient included in the study underwent a hysterectomy for the aforementioned chronic symptoms. Thus, inclusion criteria for each patient consisted of suspect diagnosis of benign uterine pathology, reproductive age and a scheduled total hysterectomy. Exclusion criteria were past or present malignancy, pregnancy, concomitant uterine infections and history of oral contraceptive, hormonal intrauterine device (IUD) or Gn-RH agonist use. The histopathological examination was used to split the patients into two study groups (UF and AM) and also served as a referral method for calculation of diagnostic accuracy of TVUS combined with SR elastography.

### 2.2. Ultrasound Examination

**Figure 1:**
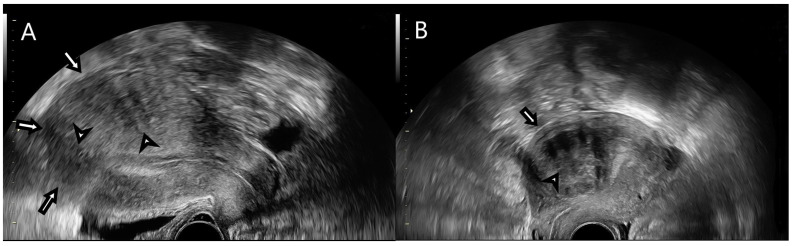
(**A**) B-mode transvaginal ultrasound features of uterine adenomyosis (highlighted by the arrows): enlarged globular uterus, fan-shaped shadowing and myometrial cysts. (**B**) B-mode transvaginal ultrasound features of uterine fibroids (highlighted by the arrows): well-defined round lesion within the myometrium with inner fan-shaped shadowing.

TVUS and SR elastography were performed by a single trained examinator with Voluson E8 BT18, Voluson E10 BT16 and Voluson E10 BT20 ultrasound systems (General Electric Healthcare, Chicago, IL, USA), using a RIC5-9-D 9 MHz vaginal probe. Two-dimensional ultrasound examination of the uterus and identification of suspected lesions were initially performed (Figure 1), and MUSA group criteria were applied for the identification of UF and AM [10].

Patients were scheduled for a hysterectomy between days 8 and 14 of menstrual cycle and 2D TVUS and SR elastography were performed less than 24 h preoperatively. In B-mode, UF features were represented by a well-defined, round lesion within the myometrium or in its surrounding vicinity, with shadows at the edge of the lesion or inner fan shaped shadowing, with symmetrical, heterogeneous, hypoechoic/hyperechoic characteristics and circumferential blood flow. For AM, B-mode ultrasonographic findings were ill-defined lesion, echogenic striations or nodules, myometrial cysts, cystic striations, interrupted junctional zone, enlarged globular uterus, fan-shaped shadowing and translesional blood flow [10].

### 2.3. Strain Ratio Elastography Analysis

**Figure 2:**
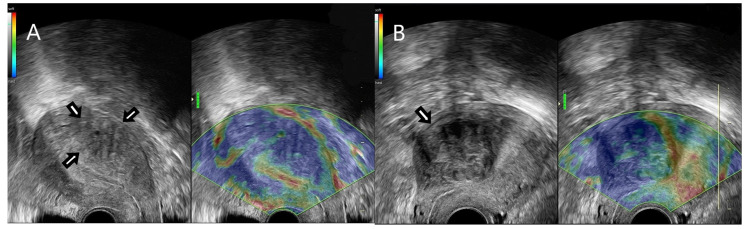
(**A**) Strain ratio elastography assessment of a real time dual mode TVUS image depicting an adenomyotic lesion. (**B**) Strain ratio elastography assessment of a real-time dual-mode TVUS image depicting a uterine fibroid.

SR assessment was achieved by using real-time dual-mode scanning after 2D ultrasound examination of the uterus and identification of suspected lesions (Figure 2).

**Figure 3:**
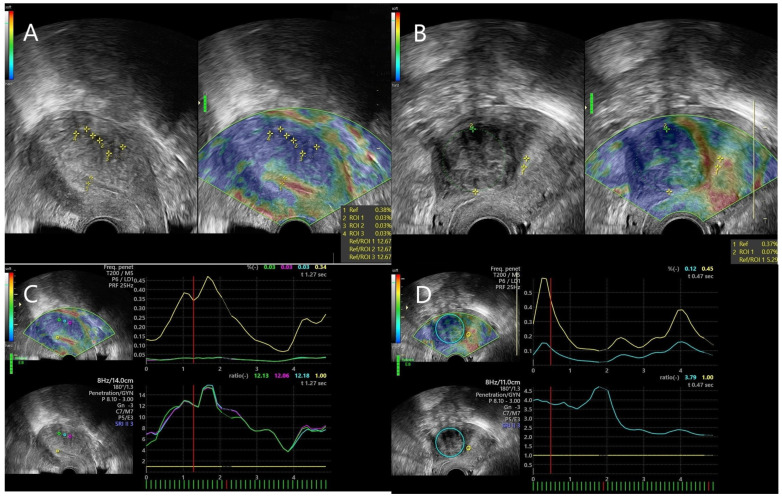
(**A**) ROI placement for comparison of tissular stiffness between healthy referral tissue and adenomyotic tissue. (**B**) ROI placement for comparison of tissular stiffness between healthy referral tissue and uterine fibroid. (**C**) Graphic representation of the elastogram obtained from an adenomyotic lesion: the line plots depict the strain ratio value of the lesional tissue (green, blue and purple lines) in comparison with the one of the referral tissue (yellow line), in accordance with standard qualitative criteria, as implied by the elastography software (**D**) Graphic representation of the elastogram obtained from a uterine fibroid: the line plots depict the strain ratio value of the lesional tissue (blue line) in comparison with the one of the referral tissue (yellow line), in accordance with standard qualitative criteria, as implied by the elastography software.

The elastogram of the suspected lesions was visualized in real time, parallelly/next to the B-mode image. In order to evaluate strain ratio images, we applied external compression by using the ultrasound probe, thus producing deformation, which was measured as strain. Three cycles of gentle compression and relaxation were performed. In the elastogram image, the color map was ranged in four colors, namely red, yellow, green and blue. Red and yellow were interpreted as soft tissue, green was considered moderately stiff tissue and blue was designed as stiff tissue. Tissue stiffness was compared with the adjacent endometrium. In consequence, the strain values and color map were relative indicators of tissue stiffness, with mean and maximum strain ratio values being calculated temporally over the compression cycles after setting the regions of interest (ROI). ROIs were represented by referral tissue (normal endometrium) and target tissue (lesions) (Figure 3).

The first ROI (ROI1) was settled as referral tissue, and the other ROIs represented the target/lesional tissue. These ROIs ranged between 5 mm and 5 cm. Three strain ratio measurements were obtained for each lesion, with similar numbers, and the mean of these measurements was calculated in each case.

### 2.4. Statistical Analysis

Statistical analysis was conducted with the help of GraphPad Prism 9.0.2 software. Descriptive statistics were conducted for quantitative variables, presented as mean and standard deviation (SD), and the Shapiro–Wilk test was used to assess their distribution. Mean comparison was performed by using the Mann–Whitney non-parametric test for non-Gaussian distributed variables (age, body mass index (BMI), and max SR values), and the unpaired t-test was applied for normally distributed variables (mean SR). Diagnostic sensitivity of elastography was calculated by dividing number of true positives to the total number of patients with confirmed diagnoses, whereas specificity implied a ratio between number of true negatives and total number of patients with confirmed diagnoses. Associations between frequency of TVUS criteria which were encountered in both adenomyosis and uterine fibroids and histology results were assessed by using Chi square tests; and odds ratio (OR), sensitivity and specificity values were calculated in each case. In order to assess the ability of elastography to differentiate adenomyosis from uterine fibroids, receiver operator characteristics (ROCs) analysis was conducted, and area under the curve (AUC) values, as well as cutoff values, with their corresponding sensitivity and specificity, were calculated for both SR mean and SR max values. Significance threshold was established at *p* < 0.05, corresponding to a confidence interval (IC) of 95%.

## 3. Results

Out of the 79 patients enrolled in the study, adenomyosis was histologically confirmed in 25 of them, whereas uterine fibroids were found in 53 subjects. One case was excluded due to a concomitant microscopic diagnosis of cervical infiltrative squamous cell carcinoma. The prevalence of MUSA group criteria among patients with histologically confirmed uterine fibroids and adenomyosis is visualized in Table 1 and Table 2, respectively.

In the case of uterine fibroids, the most common features identified upon TVUS were the presence of a well-defined round-shaped lesion with circumferential blood flow, located inside the myometrium and having a heterogeneous aspect. Adenomyotic lesions were, on the other hand, frequently associated with a globally enlarged uterus and presented in most cases echogenic striations or nodules. A comparison between frequency of commonly encountered MUSA group criteria (well-defined round lesion, fan-shaped shadowing, enlarged globular uterus, and heterogeneous lesion) and histological results is presented in Table 3. Chi square tests were applied to assess an association between those criteria and histological results. Well-defined rounded lesion characteristics represented the only criterion that was significantly able to distinguish the two uterine pathologies, in favor of uterine fibroids (*p* < 0.01). However, this TVUS criterion presented poor sensitivity and specificity parameters, when taking histology as a referral diagnosis (12% and 35.85%, respectively).

The two groups were similar in terms of age and BMI, as depicted in Table 4.

**Figure 4:**
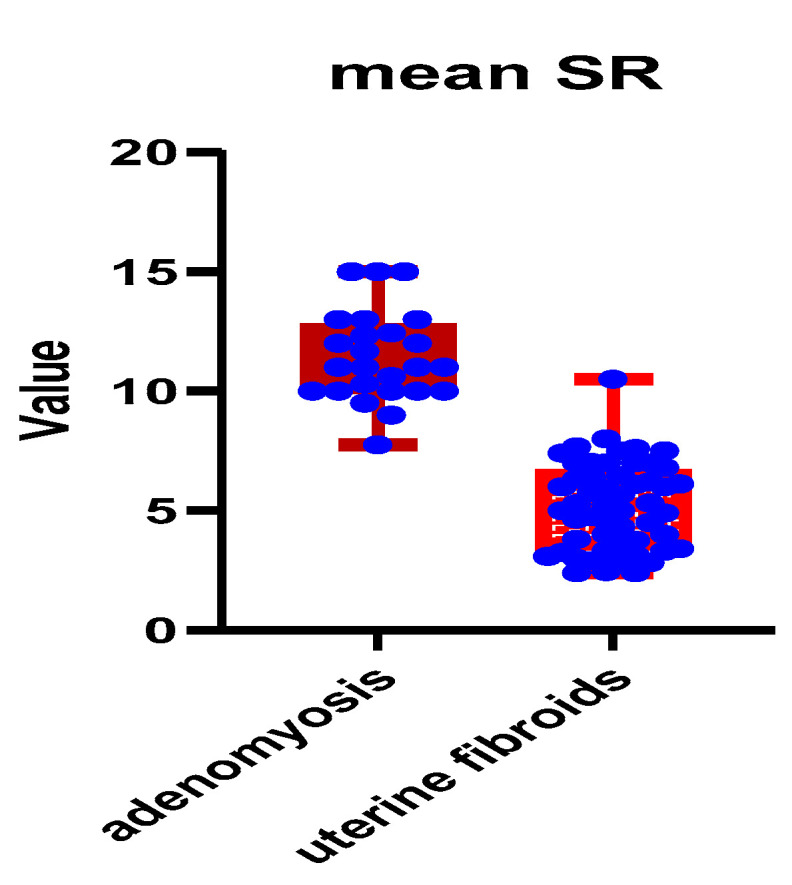
Distribution of mean SR values among the two study groups. Legend: SR = strain ratio.

**Figure 5:**
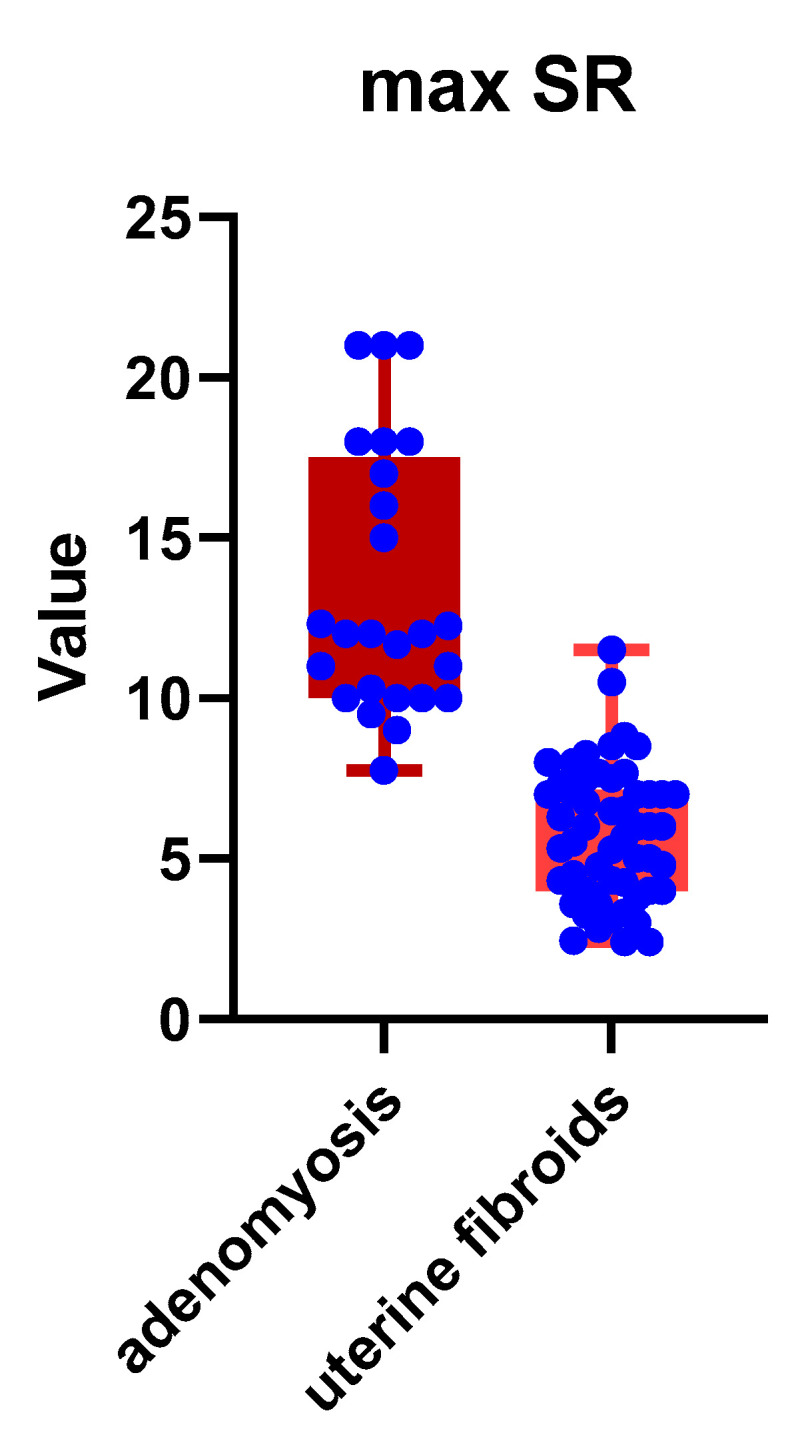
Distribution of max SR values among the two study groups. Legend: SR = strain ratio.

In accordance with elastography findings, a comparison of strain ratio scores between the two benign pathologies was conducted, which revealed significantly higher mean and max SR values in patients with microscopically confirmed adenomyosis (Table 3): 11.42 ± 1.87 SD versus 5.20 ± 1.81 SD (*p* < 0.001) and 13.43 ± 4.10 versus 5.78 ± 2.08, respectively (*p* < 0.001). Distribution of mean and max SR values among the two study groups is depicted in Figure 4 and Figure 5, respectively.

Comparison of elastography findings with the histopathological results highlighted a diagnosis sensitivity of 86.2% and a specificity of 91.37% for adenomyosis. The same parameters were calculated for uterine fibroids, which presented a higher diagnostic sensitivity (90.56%) and specificity (96.15%).

**Figure 6:**
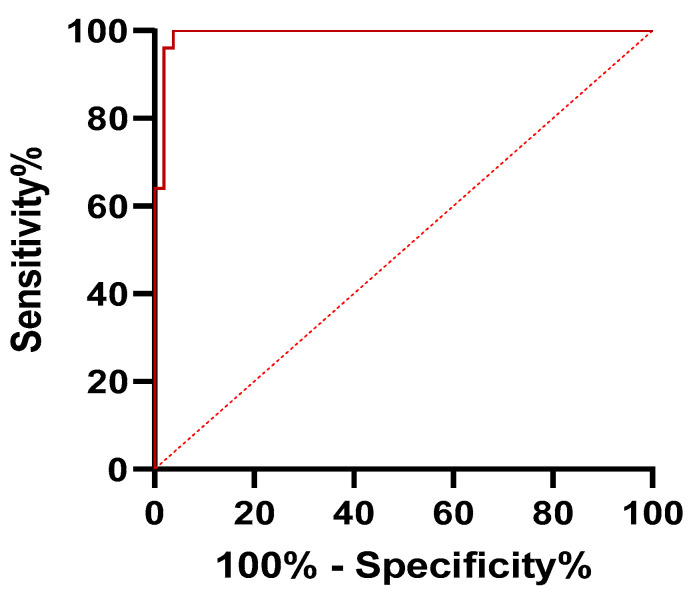
Receiver operator characteristics (ROCs) curve of mean SR values for differential diagnosis of adenomyosis from uterine fibroids.

**Figure 7:**
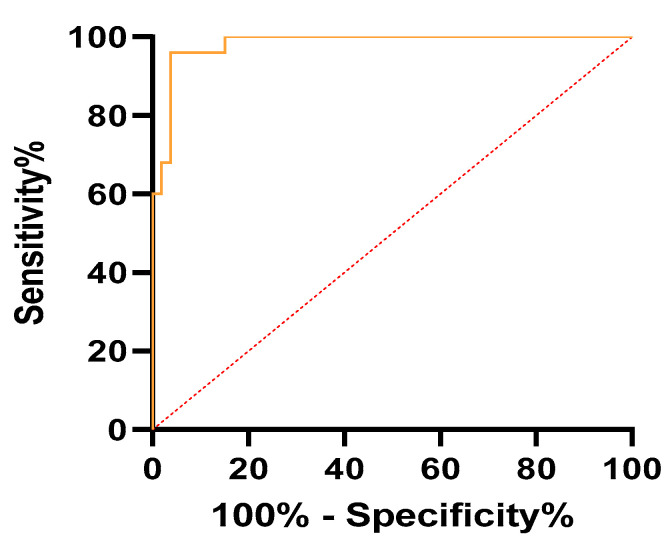
Receiver operator characteristics (ROCs) curve of max SR values for differential diagnosis of adenomyosis from uterine fibroids.

ROC analysis revealed values of AUC of 0.99 for mean SR and 0.98 for max SR, respectively (*p* < 0.001). A cutoff value of 7.71 for mean SR corresponded to a sensitivity of 100% and a specificity of 96.23%. In the case of max SR, a cutoff value of 8.91 implied a sensitivity of 96% and a specificity of 96.23%. These results are described in Table 5. Moreover, ROC curves for SR mean and SR max are represented in Figure 6 and Figure 7.

## 4. Discussion

Combined use of ultrasound and elastography, commonly referred to as sonoelastography, has successfully been used as a diagnostic tool in various pathologies, such as breast tumors, chronic liver disease, lung lesions or prostate cancer [16,17,18,19]. Application of TVUS combined with elastography in gynecology has been highlighted by studies, proving its efficacy in diagnosing and differentiating cervical, uterine wall and endometrial lesions [20,21,22]. Its main benefits over other non-invasive imagistic methods, such as computer tomography (CT) and magnetic resonance imaging (MRI), include lower costs, lack of radiation (as opposed to CT) and shorter examination time [22]. Real-time SWE has emerged as a valuable addition to conventional transvaginal ultrasound in increasing diagnostic accuracy of adenomyosis, but its ability to differentiate benign uterine pathologies is questionable [13,23]. Still, sensitivity and specificity of SWE combined with TVUS surpasses the one reported in various studies for TVUS alone [6,23]. SE, otherwise known as compression elastography, seems, on the other hand, not only to be as precise as MRI in identifying benign uterine lesions, but its utility in providing a reliable differential diagnosis between AM, UF and normal myometrium has also been highlighted [24].

Our study tried to establish the diagnostic ability of strain ratio elastography in two benign uterine disorders, through comparison with histopathological examinations, which still remains the diagnostic gold standard in both pathologies [6,25]. The comparison of pathological lesions was conducted in each case with the healthy tissue, which served as referral, with the values obtained by us representing tissue stiffness as related to healthy endometrium. Moreover, our inclusion and exclusion criteria were very strict in terms of age (pre-menopause), history of hormonal therapy and infection presence, which are all known to alter stiffness of healthy endometrium [26,27]. Furthermore, our reference in each case was the microscopic examination from the hysterectomy specimen. Thus, we did not include a control group of patients without any type of uterine disease in our study. Most of the studies focusing on TVUS combined with SE have included a control group and revealed significant differences in patients with AM or UF as opposed to healthy counterparts. A significant increase in stiffness in patients with AM was reported by most authors, exceeding the SR values of UF and even more surpassing the ones of controls [20,28]. These findings are consistent with our results, but mean and max SR values from our study were higher than those obtained in other studies. Frank et al., on the other hand, revealed a contradictory, lower lesion index for AM as opposed to normal endometrium [26].

Diagnostic sensitivity and specificity of adenomyosis calculated in our study were higher than the one reported in most studies for TVUS [29]. Stoelinga et al. obtained higher sensitivity and specificity values for adenomyoma (91% and 97%) and lower numbers for the same parameters in the case of uterine fibroids (88% and 95%) than those found in our study, but their frame of reference was represented by MRI [24]. The same authors drew the attention in an older study towards a lack of agreement between strain elastography and histology, as opposed to MRI [30]. Slightly lower sensitivity and specificity were obtained for adenomyosis in another study which compared SWE with histology (89.7% and 92.9%) [23]. Cutoff values for mean and maximum SR obtained in our study were compellingly bigger than those reported in another research [28], in relation to higher AUC, sensitivity and specificity parameters as well. Still, the variation in diagnostic accuracy is also highly dependent upon the technique used [12], as well as upon the type of ultrasound machine and probe that carried out the imagistic acquisitions. Furthermore, features of ultrasound software influence the choice of SE or SWE, as well as the color map and measurements.

Examinators’ experience, as well as their number and miscellaneous training levels, can also impact the validity of studies conducted in a similar fashion to the current one. TVUS interpretation is highly subjective [29], which explains the variation in obtaining the most appropriate images for applying strain ratio elastography. Factors influencing accuracy of TVUS include age, especially correlated with menopause status, parity, obesity or previous endometrial biopsy [31]. The reported variation in TVUS sensitivity and specificity on adenomyosis and uterine fibroids cannot be overseen, as proven by one review and two meta-analyses studies [2,15,32]. Still, subjective evaluation and measurements conducted with TVUS might be superior to the use of objective methods, as concluded by a study which compared the accuracy of the two methods in assessing myometrial invasion in patients with endometrial cancer [33]. Thus, the examinator can enrich the accuracy of TVUS. There are few studies that have analyzed differences in imaging and diagnosis conducted by various examinators, with data being limited to one study that emphasizes the lack of inter-observer variability, independently of their experience [24]. However, most of the researches conducted on the matter involved a unique, experienced examinator [20,28]. Moreover, elastography skills can be acquired in a short amount of time, but they require a solid ultrasound-training background [21]. Hence, the examinator’s experience with TVUS greatly influences the elastogram and SR values.

The main limitation of this study is the low number of cases, the unicentric design of the study and the acquisition of images by a single examinator. These aspects raise the need for further validation on researches involving larger population samples, from different geographical areas, and point out the lack of information regarding the impact of the examinator’s experience on diagnostic accuracy. The number of patients and inter-observer variability could both be responsible for variation of diagnostic sensitivity and specificity values in case of TVUS combined with SE. Moreover, the current study did not include any patients undergoing conservative treatment, as a comparison with histology was performed. Furthermore, a sonoelastography follow-up of patients treated with hormonal therapy could be an interesting research topic.

Given the few available data in the literature regarding the value of sonoelastography in the diagnosis of AM and UF, and the even scarcer information related to its use for differentiating AM from UF, our study represents a valuable addition on the aforementioned subjects. One of the strengths of this study was the comparison with histology, which remains the point of reference for the diagnosis of these two benign uterine pathologies. Besides the comparison with histological findings, the focus on the differential diagnosis of AM and UF enriches current knowledge regarding ability of SE to accurately reproduce variation in lesion stiffness. Our results confirm previous findings and support the reliable use of SE in the diagnosis of AM and UF.

## 5. Conclusions

According to our study, TVUS combined with SE presents high sensitivity and specificity in diagnosing AM and UF is are able to distinguish their peculiar lesion character, by assessing different tissular stiffness. AM presented mean and maximal tissular stiffness that significantly exceeded the one of UF. The obtained results are in accordance with most of the research studies found in the literature conducted in similar settings, but the validity of our findings is hindered by the small number of enrolled patients and dependent on the ultrasound technology characteristics. Further studies on larger populations and involving multiple examinators and ultrasound techniques could bring more precise insights on the value of sonoelastography in the diagnosis of AM and UF.

## Figures and Tables

**Table 1:** Prevalence of MUSA features among histologically confirmed uterine fibroids, as identified upon TVUS.

Uterine Fibroids—MUSA Features	
Well-defined round lesion	64%
Within the myometrium	58%
Nearby the myometrium	43%
Shadows at the edge of the lesion	24%
Inner fan-shaped shadowing	47%
Symmetrical	53%
Heterogeneous	55%
Hypoechoic/hyperechoic	47%
Circumferential blood flow	66%

**Table 2:** Prevalence of MUSA features among histologically confirmed adenomyosis, as identified upon TVUS.

Adenomyosis—MUSA Features	
Ill-defined lesion	52%
Echogenic striations or nodules	60%
Myometrial cysts	48%
Cystic striations	56%
Interrupted junctional zone	52%
Enlarged global uterus	68%
Fan-shaped shadowing	48%
Translesional blood flow	20%

**Table 3:** Chi square tests for assessment of association between frequency of commonly encountered MUSA group criteria and histological results. Legend: CI = confidence interval, MUSA group = Morphological Uterus Sonographic Assessment Group and OR = odds ratio.

MUSA Group Criteria		Adenomyosis (%)	Uterine Fibroids (%)	*p*-Value	OR (95% CI for OR)	Sensitivity % (95% CI)	Specificity % (95% CI)
Well-defined, round lesion	Yes	12	64.15	<0.01	0.07 (0.02–0.29)	12 (4.16–29.96)	35.85 (24.3–49.31)
	No	88	35.84				
Fan shaped shadowing	Yes	48	47.16	0.94	1.03 (0.39–2.69)	48 (30.03–66.5)	52.83 (39.66–65.62)
	No	52	52.83				
Enlarged globular uterus	Yes	68	58.49	0.42	1.50 (0.56–4.26)	68 (48.41–82.79)	41.51 (29.26–54.91)
	No	32	41.50				
Heterogeneous lesion	Yes	60	54.71	0.66	1.24 (0.47–3.04)	60 (40.74–76.6)	45.28 (32.66–58.55)
	No	40	45.28				

**Table 4:** Comparison of baseline and strain ratio characteristics between the two study groups Legend: SD = standard deviation, BMI = body mass index and SR = strain ratio.

Parameter (Mean ± SD)	Adenomyosis (*n* = 25)	Uterine Fibroids (*n* = 53)	*p*-Value
Age	45 ± 4.9	44 ± 5.6	0.552
BMI	25.88 ± 6.22	26.15 ± 4.02	0.266
Mean SR	11.42 ± 1.87	5.20 ± 1.81	<0.001
Max SR	13.43 ± 4.10	5.78 ± 2.08	<0.001

**Table 5:** Receiver operator characteristics (ROCs) analysis of mean and maximum SR values for distinguishing adenomyosis from uterine fibroids.

Parameter	AUC (95% CI)	Cutoff	Sensitivity (95% CI)	Specificity (95% CI)	*p*-Value
mean SR	0.99 (0.97–1)	>7.71	100% (86.68–100%)	96.23% (87.25–99.33%)	<0.001
max SR	0.98 (0.96–1)	>8.91	96% (80.46–99.79%)	96.23% (87.25–99.33%)	<0.001

## Data Availability

Data are made available from the corresponding author upon reasonable request.
